# Pre- and Post-Slaughter Methodologies to Estimate Body Fat Reserves in Lactating Saanen Goats

**DOI:** 10.3390/ani11051440

**Published:** 2021-05-18

**Authors:** Leonardo Sidney Knupp, Mondina Francesca Lunesu, Roberto Germano Costa, Mauro Ledda, Sheila Nogueira Ribeiro Knupp, Marco Acciaro, Mauro Decandia, Giovanni Molle, Ana Helena Dias Francesconi, Antonello Cannas

**Affiliations:** 1 Departamento de Zootecnia, Universidade Federal da Paraíba, Rodovia BR 079 km 12, Areia 58397-000, Brazil; LeonardoKnupp@hotmail.com; 2 Dipartimento di Agraria, Università degli Studi di Sassari, Viale Italia 39, 07100 Sassari, Italy; mflunesu@uniss.it (M.F.L.); france@uniss.it (A.H.D.F.); cannas@uniss.it (A.C.); 3 Dipartimento di Medicina Veterinaria, Università degli Studi di Sassari, Via Viena 2, 07100 Sassari, Italy; vetleddamauro@gmail.com; 4 Centro de Saúde e Tecnologia Rural, Universidade Federal de Campina Grande, Av. Universitária s/n, Patos 58708-110, Brazil; sheilanribeiro@hotmail.com; 5 Agricultural Research Agency of Sardinia—AGRIS Sardegna, Loc. Bonassai, 07100 Sassari, Italy; macciaro@agrisricerca.it (M.A.); mdecandia@agrisricerca.it (M.D.); gmolle@agrisricerca.it (G.M.)

**Keywords:** body condition score, body measurements, fat depots, goats, prediction equation, ultrasound

## Abstract

**Simple Summary:**

In this study, we present the results of a trial on which we compared pre- and post-slaughter methodologies to estimate body fat reserves in dairy goats. Our results evidenced that fat thickness measured with ultrasound in the perirenal region was the best pre-slaughter measurement for estimating fat reserves in lactating Saanen goats, whereas empty body weight and hot carcass weight were the best post-slaughter predictors for estimating fat reserves. Body condition score could be a useful tool, but it seems that it needs to be re-evaluated to predict adequately fat depots in lactating Saanen goats.

**Abstract:**

This work aimed to compare pre- and post-slaughter methodologies to estimate body fat reserves in dairy goats. Twenty-six lactating Saanen goats ranging from 43.6 to 69.4 kg of body weight (BW) and from 1.84 to 2.96 of body condition score (BCS; 0–5 range) were used. Fifteen pre-slaughter and four post-slaughter measurement values were used to estimate the weight of fat in the omental (OM), mesenteric (MES), perirenal (PR), organ (ORG), carcass (CARC), and non-carcass components (NC) and total (TOT, calculated as the sum of CARC and NC) depots in goats. The pre-slaughter measurements were withers height; rump height; rump length; pelvis width; chest depth; shoulder width; heart girth; body length; sternum height; BW; BCS assessed in the lumbar (BCSl) and sternal (BCSs) regions; and fat thickness measured by ultrasound in the lumbar (FTUSl), sternal (FTUSs), and perirenal (FTUSpr) regions. The post-slaughter measurements were hot carcass weight (HCW), empty body weight (EBW), and fat thickness measured by digital caliper in the lumbar (FTDCl) and sternal (FTDCs) regions. Linear and multiple regressions were fit to data collected. BW, BCS (from lumbar and sternal regions), all somatic measurements, and fat thickness measured by ultrasound in the lumbar and sternal regions were not adequate to estimate the weight of total fat in lactating Saanen goats (*R*^2^ ≤ 0.55). The best pre-slaughter and post-slaughter estimators of OM, MES, PR, ORG, NC, and TOT fat were FTUSpr and EBW, respectively. Among pre- and post-slaughter measurements, BCSl (*R*^2^ = 0.63) and HCW (*R*^2^ = 0.82) provided the most accurate predictions of CARC fat, respectively. Multiple regression using the pre-slaughter variables FTUSpr, BW, and BCSl yielded estimates of TOT fat with an *R*^2^ = 0.92 (RSD = 1.14 kg). On the other hand, TOT fat predicted using the post-slaughter variables HCW and FTDCs had an *R*^2^ = 0.83 (RSD = 1.41 kg). These results confirm that fat reserves can be predicted in lactating Saanen goats with high precision using multiple regression equations combining in vivo measurements.

## 1. Introduction

In most goat production systems under harsh conditions, the ability of the animal to retain and mobilize body reserves is of considerable importance in determining goat productivity and survival. Such relevance is due to the fact that the nutritional status of goats fluctuates throughout the year [1] because of changes in the amount and quality of nutrients in the diet [2] and physiological state of the animal [3]. Accurate and precise determination of nutritional status in lactating goats is important to avoid depletion of energy stored and to minimize tissue protein mobilization, thus increasing milk production [2].

The nutritional status of animals can be estimated by direct and indirect methods. The comparative slaughter is the most accurate direct method, but it is expensive, because at least half of the carcass is lost [4], it is destructive and laborious, and it does not allow for the use of the same animal more than once [5]. Therefore, indirect methods are preferable because most of them are not complex and can be applied to live animals [6].

Throughout the years, researchers have developed many indirect methods to estimate nutritional status, such as body weight (BW) and body measurements [7,8], body condition score (BCS, [9,10]), urea space [11], adipocyte diameter [12], real-time ultrasonography [13], computed tomography [1], dual-energy X-ray absorptiometry, and magnetic resonance imaging [6]. Some of these methods are very expensive and difficult to use in many farm animals. Others, such as BCS and body measurements, have basically no cost and can be performed in experimental and field conditions.

The BCS method was developed by Russel et al. [10] for meat lambs, which accumulate fat in the subcutaneous region, whereas it might not be appropriate for dairy goats, which deposit body fat mostly as visceral fat [1,14]. In lactating Alpine does, Ngwa et al. [2] noted that the amount of fat in non-carcass components (visceral and renal fat) is almost twice that in carcass and a considerable amount of internal fat is mobilized in early lactation. Härter et al. [15] developed equations to predict abdominal fat depots in pregnant non-lactating Saanen goats using ultrasound measurements of the *Longissimus* muscle area (LMA) and kidney fat thickness (KFT). The authors reported high coefficient of determination for non-carcass fat and total body fat (*R*^2^ = 0.77 and 0.80, respectively) when using LMA and KFT associated with BW. However, to our knowledge, there are no studies comparing different pre- and post-slaughter methodologies to estimate fat reserves in lactating Saanen goats. Thus, this work aimed to (i) study the relationship between BCS and body measurements with BW and body fat, (ii) compare pre- and post-slaughter methodologies as predictors to estimate body fat depots, and (iii) develop equations that could be used as an indicator of nutritional status in lactating Saanen goats.

## 2. Materials and Methods

### 2.1. Animals

The study was carried out using 26 mature lactating Saanen goats randomly selected from the experimental flock of Agris Research farm of Bonassai in Olmedo (Northwestern Sardinia, Italy, 40°40′16.215″ N, 8°22′0.392″ E, 32 m a.s.l.). Animals were chosen from a larger group fed a high-starch diet, homogeneous for lambing date, age (6–7 years), and milk yield. Goats were clinically healthy and had mean BW of 56.4 ± 6.8 kg. Animals were milked twice a day and had access to feed and water until slaughter. Their care and use followed the Italian national law and ethic regulations (DL. no. 116, 27/01/1992). The animal protocol described below was performed in compliance with the EU and Italian regulations on animal welfare, and all measurements were taken by personnel previously trained and authorized by the institutional authorities managing ethical issues at the University of Sassari. Experimental procedures with animals (goats) were approved by the Animal Care and Use Committee of the University of Sassari and Agris, Italy (CIBASA 10.12.2014).

### 2.2. Pre-Slaughter Measurements

#### 2.2.1. Somatic Measurements and Body Condition Score

The following somatic measurements, based on Cam et al. [8], were made on all goats 16 h before slaughter: withers height (WH), the distance between the top of the withers to the ground; rump length (RL), distance from hip to pin; rump height (RH) vertical distance from top of pelvic girdle and the ground; pelvis width (PW), distance between trochanters; chest depth (CD), the distance between the withers and the sternum; shoulder width (SW), the horizontal distance between the processes on the left and on the right shoulder blade; heart girth (HG), the smallest circumference around the animal just behind the foreleg; body length (BL), the distance between the withers and the cross; and sternum height (SH), the distance between the sternum and the ground. The measurements of WH, RH, CD, SW, and SH were taken with a Lydtin metric stick (metal tube of 80 to 230 cm length). Pelvis width was measured with a thickness compass, and RL, HG, and BL with a linear meter.

For practical reasons, i.e., for the lack of an appropriate precision scale suitable for live animals in the site of slaughtering, body weight was measured with an electronic scale immediately after slaughter (blood was collected and weighed). Two experienced workers evaluated the BCS in the lumbar and sternal region by using the Hervieu et al. [9] reference scale (0 to 5 score). In both cases, the BCS intervals were of 0.25 units. The BCS was assessed at the moment of the selection of the animals and at the end of the trial, just before slaughtering. 

#### 2.2.2. Measurement of Fat Thickness Using Ultrasound

Fat thickness was measured, simultaneously with the previous measures, using a real time MyLab One ultrasound system (Esaote S.p.A., Genova, Italy). Trichotomy was performed in the area to be measured and gel was used as a coupling agent to improve the quality of the images. Ultrasound pictures were taken twice on three different anatomical sites: (1) lumbar fat thickness (FTUSl), measured in the area of the longissimus muscle around the 13th thoracic vertebrae (last rib), by using an ultrasound probe SL3323 VET (array of 13-6 Mhz and 40-mm length; Esaote S.p.A., Genova, Italy); (2) perirenal fat thickness (FTUSpr), measured behind the 13th rib on the right side of the body using an ultrasound probe SV3513 VET (array of 10-5 Mhz and 50-mm length; Esaote S.p.A., Genova, Italy), according to Härter et al. [15]; and (3) sternal fat thickness (FTUSs), measured using an ultrasound probe SL3323 VET (array of 13-6 Mhz and 40-mm length; Esaote S.p.A., Genova, Italy) positioned perpendicularly to the third sternebra on the sternum. Images were obtained with a linear probe (transducer) of 6 Mhz and silicone acoustic attachment (standoff) for FTUSl and FTUSs measurements and an 8 Mhz convex transducer for FTUSpr measurements. The pictures were stored on a computer and, subsequently, analyzed with the software MyLab Desk™/Desk (Esaote S.p.A., Genova, Italy) to obtain the fat thickness measurements.

### 2.3. Post-Slaughter Measurements

#### 2.3.1. Slaughter Procedures, Hot Carcass, and Empty Body Weight

The animals were slaughtered under general anesthesia and exsanguinated from the jugular vein in the facilities of the Hospital of the Veterinary Department of the University of Sassari (Sassari, Sardinia, Italy). The weights of blood, head, skin, feet, tail, empty viscera (rumen–reticulum, omasum, abomasum, small intestine, and large intestine), mesentery, internal fat, liver, heart, kidneys, spleen, lungs, tongue, esophagus, trachea, and reproductive system, and hot carcass weight (HCW) were recorded. The digestive tract compartments were isolated, weighed, emptied, and weighed again. The empty body weight (EBW) was calculated by difference of live weight and the content of the gastrointestinal tract, bladder, and gallbladder empty. The fat tissue surrounding the digestive tract, omental (OM) fats, and mesenteric (MES) fats was removed, along with any associated connective tissue and weighed. Perirenal fat (PR) was removed from the kidneys and weighed. Organ fat from heart, liver, and lungs was removed from each organ and weighed together (ORG).

#### 2.3.2. Carcass Measurements

Carcasses were stored at 4 °C for 24 h in a cooler. Then, carcasses were split down the backbone with a band saw into two halves (right and left). The right half of each carcass was ribbed at the 12th and 13th thoracic vertebrae at the same anatomical points where measurements had been taken on the live animal using ultrasound. Lumbar fat thickness was measured by using a digital caliper (FTDCl). Similarly, a transversal cut was performed at the third sternebra on the sternum vertebra, and sternal fat was then measured using a digital caliper (FTDCs).

#### 2.3.3. Fat Content on Carcass and Non-Carcass Components

The left side of each carcass was frozen until subsequent determination of chemical composition, whereas all non-carcass components (digestive tract, pluck, reproductive tract, and mammary gland), including head and skin, were stored in separate polyethylene bags at −20 °C until preparation for analysis. All frozen components (carcass and non-carcass) were cut into pieces of 5–6 cm^3^ while still frozen, and then minced and ground by using a mill grinder (TC 42 Golia HP 10 HS, La Felsinea S.R., Padova, Italy). After the ground material was mixed thoroughly with a mechanical mixer (ME 30, La Felsinea S.R., Padova, Italy), samples were taken in three replicates. The samples were weighed, frozen at −80 °C, and subsequently analyzed for dry matter by liophilization (Lyolab 3000, Jouan Nordic, Allerød, Denmark). Then, samples were reground in a blender (Knifetec Mill 1095, Foss, Höganăs, Sweden) and analyzed for fat. Carcass (CARC) and non-carcass (NC) fat was determined by continuously extracting the samples with petroleum ether for 6 h by using the AOAC method 920.39 (AOAC International, 2005).

### 2.4. Statistical Analysis

The statistical analyses were performed using linear single variable with the GLM procedure of SAS software (version 9.2, SAS System Inc., Cary, NC, USA) for the weights of fat in the different depots as dependent variables (y), and BW, BCSl, BCSs, somatic measurements, FTUSl, FTUSs, FTUSpr, HCW, EBW, FTDCl, and FTDCs as independent variables (x). The variables included in the multiple regressions were selected using the REG procedure with the STEPWISE method of SAS. Since ultrasound is not so cheap and requires more time than BCS, BW, and somatic measures to be used under field conditions, additional simplified equations were developed without the use of ultrasound also using the REG procedure with the STEPWISE method of SAS.

## 3. Results

### 3.1. Pre-Slaughter Measurements

#### 3.1.1. Somatic Measurements

**Figure 1:**
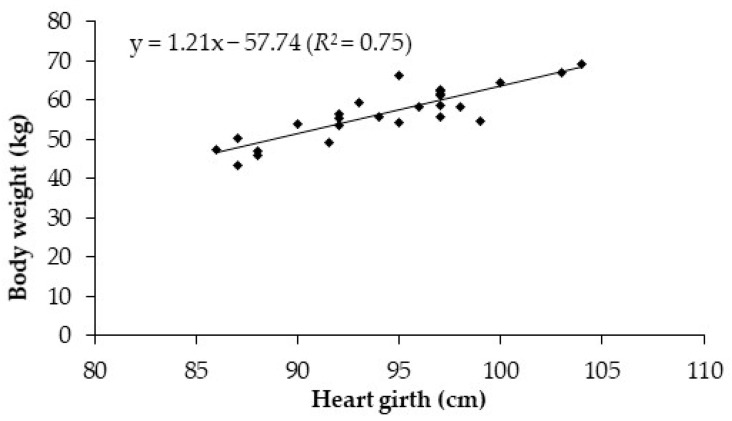
Relationship between heart girth and body weight in lactating Saanen goats.

Heart girth ranged from 86 to 104 cm, with a mean of 94 cm (Table 1). Among all somatic measurements, only HG had regression coefficients significantly different from zero (*p* < 0.05) in all fat depots analyzed. The relationship between HG and BW showed a mean HG change of 1.2 cm per unit (kg) of BW (BW = 1.2 HG − 57.7; *R*^2^ = 0.75; Figure 1). 

#### 3.1.2. Body Weight

Body weight after slaughter (summed with blood from exsanguinations) ranged from 44 to 69 kg, with a mean of 56 kg (Table 1). The regressions between the weight of fat in each of the different fat depots and BW (Table 2) had determination coefficients (*R*^2^) that varied between 0.21 for the organ (ORG) depot (RSD = 0.17 kg) and 0.58 for carcass depot (RSD = 0.86 kg). The determination coefficient for the relationship between the total weight of fat (TOT, sum of fat on carcass and non-carcass components) and BW (Table 2) was 0.55 (RSD = 2.25 kg).

#### 3.1.3. Body Condition Score

Body condition score assessed at lumbar or sternal region averaged 2.6, but sternal BCS detected a lower fatness level compared to BCSl (1.75 versus 1.84, respectively) (Table 1). The Pearson correlation between lumbar and sternal BCS was 0.852, with *p* < 0.001. The regression of sternal BCS on lumbar BCS had a non-significant intercept, with BCS sternal = 0.999 BCS lumbar. The regression equations for prediction of the weights of fat depots using both BCS, from lumbar and sternal region, had low accuracy. The *R*^2^ values for BCSl varied between 0.10 for organs and 0.63 for carcass fat, and those for BCSs varied between 0.07 for organs and 0.54 for carcass fat (Table 2). 

**Figure 2:**
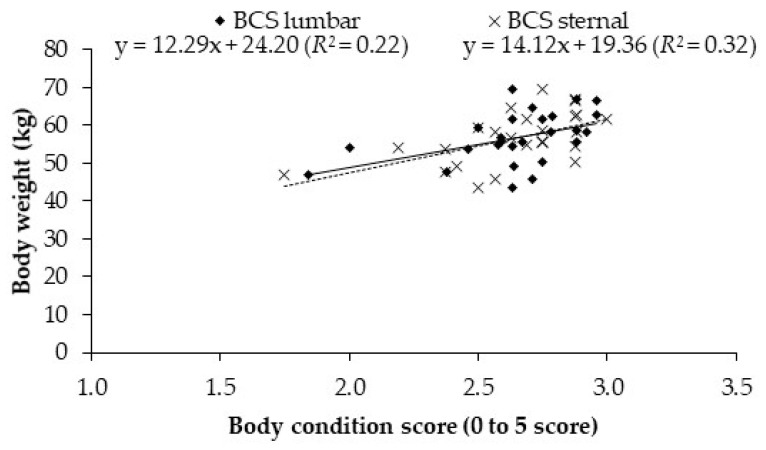
Relationship between lumbar or sternal body condition score (BCS, scale 0–5) and body weight in lactating Saanen goats.

The relationship between lumbar and sternal BCS and BW in lactating Saanen goats provided low *R*^2^ (Figure 2). For BCSl, the equation was BW (kg) = 12.29 BCSl + 24.20 (*R*^2^ = 0.22) and for BCSs, the equation was BW (kg) = 14.12 BCSs + 19.36 (*R*^2^ = 0.32).

#### 3.1.4. Ultrasound Measurements

Thickness of fat in the lumbar region measured using ultrasound ranged from 1.1 to 3.5 mm, with a mean of 2.3 mm (SD = 0.6). Fat in the sternal region was much thicker, ranging from 9.7 to 27.9 mm, with a mean of 22.7 mm (SD = 4.5). Perirenal fat was also high, ranging from 44 to 234 mm, with a mean of 121 mm (SD = 53) (Table 1). The determination coefficients of the equations were slightly lower using FTUSl, ranging between 0.10 for organ fat (RSD = 0.18 kg) and 0.33 for non-carcass fat (RSD = 1.76 kg), than using FTUSs thickness, ranging from 0.05 for organ fat (RSD = 0.19 kg) and 0.55 for carcass fat (RSD = 0.89 kg) (Table 3). Nevertheless, higher *R*^2^ were found using FTUSpr, with values ranging between 0.05 for organ fat (RSD = 0.19 kg) and 0.86 for PR fat (RSD = 0.22 kg) (Table 3).

#### 3.1.5. Multiple Regressions

To increase the accuracy of the equation that predicted fat depot using only one independent variable (Table 2, Table 3 and Table 4), we calculated multiple regressions (Table 5). The inclusion of heart girth in the regression using FTUSpr to predict omental fat improved the *R*^2^ from 0.79 to 0.85 (RSD = 0.65 and 0.57 kg, respectively), and, when predicting mesenteric fat, it improved the *R*^2^ from 0.46 to 0.62 (RSD = 0.26 and 0.23 kg, respectively). The weight of perirenal fat was best predicted by an equation that included both FTUSpr and BW, increasing the *R*^2^ value from 0.84 to 0.88 (RSD = 0.26 and 0.23 kg, respectively) compared to FTUSpr alone. The carcass fat weight was best predicted by an equation with three variables, FTUSpr, BW, and BCSl (*R*^2^ = 0.92, RSD = 0.46 kg). Similarly, non-carcass fat weight was best predicted by an equation with three variables (FTUSpr, HG, and BCSl; *R*^2^ = 0.91, RSD = 0.71 kg). The best equation to predict total fat weight included FTUSpr, BW, and BCSl (*R*^2^ = 0.92, RSD = 1.14 kg).

### 3.2. Post-Slaughter Measurements

#### 3.2.1. Hot Carcass Weight

Hot carcass weight varied between 18.1 and 30.1 kg with a mean of 24.3 kg (SD = 3.4 kg) (Table 1), corresponding to a mean killing out percentage (100 × HCW/BW) of 42.7 ± 3.0 (data not shown). The values of *R*^2^ for equations using HCW varied from 0.17 for organ fat (RSD = 0.18 kg) to 0.82 for carcass fat (RSD = 0.57 kg), with a value of 0.74 for total fat (RSD = 1.73 kg) (Table 4).

#### 3.2.2. Empty Body Weight

Empty body weight mean was 47.6 kg, varying between 36.1 and 59.9 kg (Table 1). The values of *R*^2^ for equations using EBW varied from 0.23 for organ fat (RSD = 0.17 kg) to 0.74 for carcass fat (RSD = 0.67 kg) and total fat (RSD = 1.70 kg) (Table 4).

#### 3.2.3. Digital Caliper Measurements

The mean depths of the fat measured by digital caliper in the lumbar and sternal regions were 2.1 mm (range 1.5–3.6 mm) and 22.1 mm (range 9.8–29.8 mm), respectively (Table 1). The determination coefficients of the equations using FTDCl as a predictor were all extremely low (varying between 0.01 and 0.17) and were not significant (*p* > 0.05, except ORG). Predictions using FTDCs had *R*^2^ values between 0.11 for organ fat (RSD = 0.18 kg) and 0.61 for carcass fat (RSD = 0.82 kg) (Table 4). 

#### 3.2.4. Multiple Regressions

The inclusion of FTDCs in in the equation using EBW to predict the weight of the OM fat resulted in an improvement in the accuracy (*R*^2^ value increased from 0.47 to 0.52; Table 6). In contrast, the prediction of the MES fat weight, where EBW was the best single predictor, was not improved by the addition of any other post-slaughter variables. The addition of FTDCs, in combination with EBW, increased the *R*^2^ value from 0.46 to 0.53 (RSD = 0.44 and 0.43 kg, respectively) in the equation to predict PR fat weight and from 0.68 to 0.74 (RSD = 1.20 and 1.12 kg, respectively) in the equation to predict non-carcass fat. For the prediction of organ fat, the equation obtained had a coefficient of determination very low (*R*^2^ = 0.32) with the use of the EBW and FTDCl. 

The predictions of carcass and total fat were markedly more precise than those of the internal organs. The addition of the FTDCs with HCW increased the precision from 0.82 to 0.91 (RSD = 0.57 and 0.40 kg, respectively) to predict carcass fat weight (Table 6), while the addition of the FTDCs with HCW increased the precision from 0.74 to 0.83 (RSD = 1.73 and 1.41 kg, respectively) to predict total fat weight (Table 6).

## 4. Discussion

As mentioned in the method, for practical reasons, i.e., for the lack of an appropriate precision scale suitable for live animals in the site of slaughtering, body weight was measured with an electronic scale immediately after slaughter (blood was collected and weighed). Since this technique avoided the errors associated with animal movement during weighing, it is likely that BW measurement immediately post-mortem was at least as accurate and precise as when carried out on live animals. 

Among all body dimension characters evaluated, HG was the most related trait to BW (BW = 1.2 HG − 57.7; *R*^2^ = 0.75; Figure 1). In a recent work, McGregor [16] observed a moderate correlation (*R*^2^ = 0.60) in Angora goats, with a 1 kg increase in live weight for each 1 cm increase in heart girth, which was very similar to the present work. Slippers et al. [17] reported that body weight was highly correlated with heart girth in Nguni goats (*R*^2^ > 0.88). In contrast to what observed for HG, BCS was not a good predictor to estimate live weight (Figure 2), probably because of the moderate correlation between BW and lumbar BCS (*r* = 0.50) and sternum BCS (*r* = 0.56). McGregor [16] reported a correlation of 66% between BW and lumbar BCS, corroborating that it is difficult to estimate the BW using BCS in goats. Although the level of precision obtained when using BW to predict weights of fat depots such as organ fat and omental fat was not high (*R*^2^ = 0.21 and 0.28, respectively), a higher precision was achieved when predicting carcass and total fat content (*R*^2^ = 0.58 and 0.55, respectively). 

When using the lumbar BCS method, Russel et al. [10] in Scottish Blackface ewes and Teixeira et al. [18] in Rasa Aragonesa ewes obtained *R*^2^ values close to 0.90 for BCS as a predictor of the amount of body fat. However, the distribution of body fat in goats differs appreciably from that in ewes [19]. The data of the present study in Saanen goats confirmed that subcutaneous fat deposits are not highly noticeable in the dorsal region of this species. In fact, according to Hervieu et al. [9], large amounts of accumulated fat are deposited in the sternal region in goats. Although Mendizabal et al. [12] reported that the precision to estimate total fat in Spanish Blanca Celtibérica goats using sternal BCS was much better (*R*^2^ = 0.90) than those achieved using lumbar BCS (*R*^2^ = 0.59), in the present study, sternal BCS did not estimate fat reserves satisfactorily (*R*^2^ < 0.55). Furthermore, in the same region, both ultrasound (*R*^2^ = 0.51) and digital caliper (*R*^2^ = 0.57) had low precision in the estimation of fat reserves. These differences can be attributed, at least in part, to the much greater ranges of BW and BCS (33.0 to 80.5 kg and 0.75 to 4.25, respectively) evaluated by Mendizabal et al. [12] compared to those obtained in the present work (43.6 to 69.4 kg and 1.75 to 3.00, respectively). This is plausible considering the mathematical and statistical approaches used because the regression fit of the model is dependent on the range of the dataset. The utilization of high ranges of BCS is scientifically correct but tends to overestimate the ability of the method to predict the actual body reserves and visceral fat of the animals, since it includes a range of BCS and body reserves values rarely seen in commercial goat flocks (e.g., Eknaes et al. [1] estimated a total body fat and protein content in goats in different stages of lactation raised intensively and extensively lower than that in our experiment, reported in Table 1), while a method to estimate body reserves should work within the values commonly observed in commercial flocks. Another possible explanation is that Saanen goats do not deposit fat in the lumbar or sternal region proportionally to the visceral fat depots. 

Among all somatic measurements taken, only heart girth presented a significant correlation with all fat depots. However, the determination coefficients of the equations using heart girth were consistently low, with values ranging between 0.16 for organ fat (RSD = 0.56 kg) and 0.46 for carcass fat (RSD = 0.98 kg). Differently, in Pelibuey ewes, Bautista-Díaz et al. [7] observed that abdominal circumference was the best somatic measurement taken to estimate the weights of carcass fat (*R*^2^ = 0.73), visceral fat (*R*^2^ = 0.64), and total body fat (*R*^2^ = 0.71). In fact, these results confirm that sheep, especially meat breeds, have a higher deposition of fat in the subcutaneous region, whereas dairy goat breeds deposit a major part of fat in the visceral internal cavity [14].

When estimating fat depots using ultrasound, we attained higher levels of precision when measuring the perirenal fat thickness (*R*^2^ values between 0.05 and 0.86) compared to the lumbar region (*R*^2^ values from 0.10 to 0.33) or the sternal region (*R*^2^ values from 0.05 to 0.55). These findings confirmed that the perirenal fat thickness measured with ultrasound can adequately estimate fat reserves in lactating Saanen goats (except ORG fat). In fact, in a previous study carried out on Saanen goats, Härter et al. [15] found that abdominal fat was the main energy reserve and that perirenal fat thickness measured by ultrasound was significantly correlated with BW and renal, omental, and non-carcass fat.

Considering the post-slaughter measurements evaluated in this study, we found that hot carcass weight and empty body weight were the best predictors of the amount of total fat stored by the goats (*R*^2^ = 0.74 and RSD = 1.7 kg, for both). The use of HCW or EBW removes the large effect that differences in gastrointestinal contents, which varied from 5.6 to 12.7 kg, have on BW. Similarly, Mendizabal et al. [2] found that EBW and, especially, HCW were the best post-slaughter predictors of the weights of fat depots in Spanish Blanca Celtibérica goats.

Lumbar fat thickness measured by a digital caliper was the worst predictor of the weights of individual fat depots, with *R*^2^ values lower than 0.2, likely because the very thin layer of fat that lost its firmness after cutting the muscle and, therefore, made measurements difficult. Sternal fat thickness measured by a digital caliper was not a good predictor of fat depots either, although *R*^2^ values were higher (0.11–0.61 range) compared to the lumbar region. In Spanish Blanca Celtibérica goats, Delfa et al. [20] dissected the lumbar and sternal region and found that the fat percentage of the lumbar square joint was only 15% compared to 41% of fat in the sternal triangle joint. Therefore, it is evident that the BCS scales proposed by Hervieu et al. [9] for Alpine and Saanen goats need to be re-evaluated. A BCS method based on body palpations is difficult to adopt in goats because of a lack of subcutaneous adipose tissue in this species. Firstly, it would be necessary to evaluate if there is a correlation between the fat located in the lumbar or sternal region and the total fat of the animals. If findings show a high correlation, this could mean that the BCS methods can be used to predict the body fat of dairy goats, although some adjustments might still be necessary. However, if studies show that the fat thickness located in the lumbar and sternal region is not highly correlated with the total fat, mainly located in the visceral region, then new methods should be developed. Hervieu et al. [9] confirmed that there is a significant correlation between the fat scores given by BCS and their respective fat fractions (in the lumbar and sternal regions). However, the authors did not evaluate whether this correlation also regarded the body composition as a whole. 

On the basis of the different regressions of the pre-slaughter measurements tested for each fat depot, we found that FTUSpr yielded the most precise estimates of body fat in lactating Saanen goats, with the exception of organ fat depot estimation, and the addition of BW and BCSl substantially improved the precision of the estimates of total body fat (*R*^2^ increasing from 0.62 to 0.92). We hypothesized that there is a wide variability of body fat on equal BCS (low precision of estimation). However, the estimation of carcass and total fat could be improved if BW and BCSl were added to FTUSpr, as shown in Table 5. These results suggest that goats were of different sizes (large and small) and possibly in some cases with similar BCS. In addition, BW was a discrete indicator of carcass and total fat and was moderately accurate indicator for MES fat; BCSl was a particularly good predictor of carcass fat and FTUSpr predicted with high accuracy omental, perirenal, and non-carcass fat. Therefore, the addition of these three variables (BW, BCSl, and FTUSpr) seems to be complementary in predicting total fat.

On the other hand, on the basis of the multiple regression analysis using post-slaughter measurements, we found that EBW was the first variable and gave the best predictions of OM, MES, PR, ORG, and NC fat depots, whereas HCW was the first variable in CARC and TOT fat. The addition of FTDCs as a second variable was helpful when estimating the fat reserves in OM, PR, NC, and TOT fat (*R*^2^ increasing from 0.74 to 0.83). These results agree with those obtained by Mendizabal et al. [12], who found that HCW and EBW were the most used post-slaughter variables to predict fat depots in Spanish Blanca Celtibérica goats, confirming the importance of these measurements to predict fat depots in goats.

When the main results obtained with the multiple regression analysis are considered, the recommended equations to be used at field level, when ultrasound is not available, might be summarized as

(1) OM: 4.79 + 0.13 × BW + 1.57 × BCSl − 0.16 × WH − 0.15 × RL (*R*^2^ = 0.55);
(2) MES: 1.57 + 0.05 × BW − 0.04 × WH (*R*^2^ = 0.56);
(3) PR: 2.79 + 0.08 × BW − 0.07 × WH − 0.09 × PW (*R*^2^ = 0.48);
(4) ORG: 1.21 + 0.02 × BW − 0.06 × CD (*R*^2^ = 0.60);
(5) CARC: −3.31 + 0.12 × BW + 3.19 × BCSl − 0.07 × RH − 0.12 × SW (*R*^2^ = 0.87);
(6) NC: 1.65 + 0.25 × BW + 2.61 × BCSl − 0.16 × WH − 0.31 × PW (*R*^2^ = 0.69);
(7) TOT: −4.21 + 0.36 × BW + 6.12 × BCSl − 0.21 × WH − 0.38 × SW (*R*^2^ = 0.79).

## 5. Conclusions

Fat thickness measured with ultrasound in the perirenal region was the best pre-slaughter measurement for estimating fat reserves in lactating Saanen goats, whereas empty body weight and hot carcass weight were the best post-slaughter predictors for estimating fat reserves. Body condition score could be a useful tool, but it seems that it needs to be re-evaluated to predict adequately fat depots in lactating Saanen goats. The best variable to predict carcass and total fat content was hot carcass weight, but methodologies able to predict weights of fat reserves in live animals are preferable for practical and economic reasons. 

## Figures and Tables

**Table 1:** Somatic measurements; body weight (BW); lumbar and sternal body condition scores (BCSl and BSs, respectively); hot carcass weight (HCW); empty body weight (EBW); lumbar, sternal, and perirenal fat thickness depth measured by ultrasound (FTUSl, FTUSs, and FTUSpr, respectively); lumbar and sternal fat thickness depth measured by digital caliper (FTDCl and FTDCs, respectively); and fat depot weights.

Item	Mean	Minimum	Maximum	Standard Deviation
Somatic measurements (cm)				
Wither height	70.9	65.0	77.0	3.4
Rump height	73.6	69.0	82.0	3.3
Rump length	21.6	17.5	28.0	3.0
Pelvis width	18.7	16.0	25.0	1.9
Chest depth	35.1	29.0	39.0	2.1
Shoulder width	18.1	13.5	24.0	2.5
Heart girth	94.2	86.0	104.0	4.8
Body length	74.7	68.0	90.0	4.7
Sternum height	35.3	28.0	44.0	3.9
BW (kg)	56.4	43.6	69.4	6.8
BCSl (scale 0–5)	2.64	1.84	2.96	0.3
BCSs (scale 0–5)	2.64	1.75	3.00	0.3
HCW (kg)	24.3	18.1	30.1	3.4
EBW (kg)	47.6	36.1	59.9	6.8
FTUSl (mm)	2.27	1.08	3.50	0.6
FTDCl (mm)	2.13	1.47	3.60	0.5
FTUSs (mm)	22.68	9.70	27.90	4.5
FTDCs (mm)	22.10	9.79	29.82	4.7
FTUSpr (cm)	1.21	0.44	2.34	0.1
Fat depot weight (kg)				
Omental	1.83	0.30	4.38	1.3
Mesenteric	0.94	0.46	1.54	0.3
Perirenal	0.72	0.07	2.02	0.6
Organ fat (heart, liver, and lungs)	0.22	0.08	1.06	0.2
Total fat (kg)				
Carcass	4.61	1.33	6.58	1.3
Non-carcass	5.56	2.16	9.38	2.1
Carcass and non-carcass	10.17	3.49	15.93	3.2

**Table 2:** Regression equations (y = a + bx), coefficient of determination (*R*^2^), and residual standard deviation (RSD) values for estimate of the different fat depot weights (kg) and the total weight of all fat combined (y variables) based on the pre-slaughter measurement values of heart girth, body weight (BW), and lumbar and sternal body condition scores (x variables).

Item ^1^	Intercept ± Standard Error	b ± Standard Error	*R* ^2^	RSD	*p*-Value
Heart girth					
OM	−9.26 ± 4.58	0.12 ± 0.05	0.20	1.17	0.023
MES	−2.96 ± 1.06	0.04 ± 0.01	0.36	0.27	0.001
PR	−3.83 ± 2.17	0.05 ± 0.02	0.16	0.56	0.046
ORG	−1.47 ± 0.67	0.02 ± 0.01	0.21	0.17	0.019
CARC	−12.49 ± 3.82	0.18 ± 0.04	0.46	0.98	0.001
NC	−19.27 ± 6.58	0.26 ± 0.07	0.37	1.68	0.001
TOT	−31.76 ± 9.95	0.44 ± 0.10	0.43	2.55	0.001
BW at slaughter					
OM	−3.82 ± 1.87	0.10 ± 0.03	0.28	1.11	0.005
MES	−0.86 ± 0.43	0.03 ± 0.01	0.43	0.26	0.001
PR	−1.89 ± 0.86	0.05 ± 0.01	0.28	0.51	0.005
ORG	−0.49 ± 0.29	0.01 ± 0.005	0.21	0.17	0.020
CARC	−3.65 ± 1.45	0.15 ± 0.02	0.58	0.86	<0.001
NC	−6.55 ± 2.57	0.21 ± 0.04	0.49	1.52	<0.001
TOT	−10.20 ± 3.80	0.36 ± 0.07	0.55	2.25	<0.001
BCS lumbar (scale 0–5)					
OM	−3.95 ± 2.38	2.21 ± 0.89	0.20	1.17	0.022
MES	−0.42 ± 0.63	0.52 ± 0.24	0.17	0.31	0.038
PR	−1.73 ± 1.12	0.93 ± 0.42	0.17	0.55	0.037
ORG	−0.38 ± 0.37	0.23 ± 0.14	0.10	0.18	0.116
CARC	−5.79 ± 1.64	3.94 ± 0.62	0.63	0.81	<0.001
NC	−6.30 ± 3.57	4.50 ± 1.34	0.32	1.76	0.003
TOT	−12.08 ± 5.07	8.44 ± 1.91	0.45	2.50	0.001
BCS sternal (scale 0–5)					
OM	−3.08 ± 2.37	1.87 ± 0.89	0.16	1.20	0.047
MES	−0.49 ± 0.60	0.55 ± 0.22	0.20	0.30	0.023
PR	−1.45 ± 1.10	0.83 ± 0.42	0.14	0.56	0.057
ORG	−0.28 ± 0.37	0.19 ± 0.14	0.07	0.19	0.183
CARC	−4.76 ± 1.76	3.55 ± 0.66	0.54	0.90	<0.001
NC	−4.95 ± 3.59	3.99 ± 1.35	0.27	1.82	0.007
TOT	−9.71 ± 5.21	7.54 ± 1.96	0.38	2.64	0.001

^1^ OM = omental fat; MES = mesenteric fat; PR = perirenal fat; ORG = organ fat (heart, liver, and lungs); CARC = carcass fat; NC = non-carcass fat; TOT = total fat depot (TOT = CARC + NC).

**Table 3:** Regression equations (y = a + bx), coefficient of determination (*R*^2^), and residual standard deviation (RSD) values for estimate of the different fat depot weights (kg) and the total weight of all fat combined (y variables) based on the pre-slaughter measurement values lumbar, sternal, and perirenal fat thickness depths measured by ultrasound (FTUSl, FTUSs, and FTUSpr, respectively) (x variables).

Item ^1^	Intercept ± Standard Error	b ± Standard Error	*R* ^2^	RSD	*p*-Value
Lumbar fat thickness (FTUSl)					
OM	−0.46 ± 0.92	1.04 ± 0.39	0.23	1.16	0.012
MES	0.37 ± 0.24	0.27 ± 0.10	0.23	0.30	0.012
PR	−0.38 ± 0.42	0.50 ± 0.18	0.25	0.53	0.008
ORG	−0.003 ± 0.14	0.10 ± 0.06	0.10	0.18	0.120
CARC	2.07 ± 0.92	1.13 ± 0.39	0.27	1.16	0.007
NC	1.10 ± 1.40	2.01 ± 0.59	0.33	1.76	0.002
TOT	3.16 ± 2.23	3.14 ± 0.95	0.32	2.81	0.002
Sternal fat thickness (FTUSs)					
OM	−1.81 ± 1.05	0.16 ± 0.05	0.35	1.06	0.001
MES	0.17 ± 0.29	0.03 ± 0.01	0.24	0.29	0.011
PR	−1.01 ± 0.48	0.08 ± 0.02	0.37	0.48	0.001
ORG	0.02 ± 0.19	0.01 ± 0.01	0.05	0.19	0.278
CARC	−0.04 ± 0.88	0.21 ± 0.04	0.55	0.89	<0.001
NC	−1.11 ± 1.57	0.30 ± 0.07	0.44	1.59	0.001
TOT	−1.15 ± 2.33	0.51 ± 0.10	0.51	2.36	<0.001
Perirenal fat thickness (FTUSpr)					
OM	−0.53 ± 0.26	2.23 ± 0.22	0.81	0.57	<0.001
MES	0.48 ± 0.11	0.45 ± 0.09	0.49	0.24	<0.001
PR	−0.40 ± 0.10	1.06 ± 0.09	0.86	0.22	<0.001
ORG	0.14 ± 0.09	0.08 ± 0.07	0.05	0.19	0.283
CARC	2.88 ± 0.46	1.64 ± 0.49	0.42	1.01	0.001
NC	1.94 ± 0.51	3.44 ± 0.43	0.73	1.11	<0.001
TOT	4.82 ± 0.93	5.07 ± 0.78	0.63	2.03	<0.001

^1^ OM = omental fat; MES = mesenteric fat; PR = perirenal fat; ORG = organ fat (heart, liver, and lungs); CARC = carcass fat; NC = non-carcass fat; TOT = total fat depot (TOT = CARC + NC).

**Table 4:** Regression equations (y = a + bx), coefficient of determination (*R*^2^), and residual standard deviation (RSD) values for estimate of the different fat depot weights (kg) and the total weight of all fat combined (y variables) based on the post-slaughter measurement values hot carcass weight (HCW), empty body weight (EBW), and lumbar and sternal fat thickness depths measured by a digital caliper (FTDCl and FTDCs, respectively) (x variables).

Item ^1^	Intercept ± Standard Error	b ± Standard Error	*R* ^2^	RSD	*p*-Value
Hot carcass weight (HCW)					
OM	−4.00 ± 1.39	0.24 ± 0.06	0.43	0.99	0.001
MES	−0.81 ± 0.31	0.07 ± 0.01	0.58	0.22	<0.001
PR	−1.95 ± 0.65	0.11 ± 0.03	0.42	0.46	0.001
ORG	−0.32 ± 0.25	0.02 ± 0.01	0.17	0.18	0.037
CARC	−3.55 ± 0.80	0.34 ± 0.03	0.82	0.57	<0.001
NC	−5.89 ± 1.83	0.47 ± 0.07	0.63	1.30	<0.001
TOT	−9.44 ± 2.43	0.81 ± 0.10	0.74	1.73	<0.001
Empty body weight (EBW)					
OM	−4.30 ± 1.34	0.13 ± 0.03	0.47	0.95	0.001
MES	−0.86 ± 0.30	0.04 ± 0.01	0.61	0.21	<0.001
PR	−2.08 ± 0.63	0.06 ± 0.01	0.46	0.44	0.001
ORG	−0.42 ± 0.24	0.01 ± 0.01	0.23	0.17	0.012
CARC	−3.20 ± 0.95	0.16 ± 0.02	0.74	0.67	<0.001
NC	−6.44 ± 1.69	0.25 ± 0.03	0.68	1.20	<0.001
TOT	−9.64 ± 2.40	0.42 ± 0.05	0.74	1.70	<0.001
Lumbar fat thickness (FTDCl)					
OM	1.52 ± 1.12	0.16 ± 0.51	0.01	1.31	0.760
MES	0.75 ± 0.29	0.10 ± 0.13	0.02	0.33	0.473
PR	0.72 ± 0.52	0.01 ± 0.24	0.01	0.61	0.976
ORG	−0.10 ± 0.15	0.15 ± 0.07	0.17	0.18	0.036
CARC	3.42 ± 1.11	0.56 ± 0.51	0.05	1.29	0.279
NC	4.65 ± 1.82	0.45 ± 0.83	0.01	2.12	0.591
TOT	8.07 ± 2.86	1.02 ± 1.31	0.02	3.32	0.444
Sternal fat thickness (FTDCs)					
OM	−1.97 ± 1.03	0.17 ± 0.04	0.37	1.03	0.001
MES	0.09 ± 0.29	0.04 ± 0.01	0.28	0.29	0.005
PR	−1.09 ± 0.47	0.08 ± 0.02	0.40	0.47	0.001
ORG	−0.08 ± 0.18	0.01 ± 0.01	0.11	0.18	0.099
CARC	−0.34 ± 0.82	0.22 ± 0.04	0.61	0.82	<0.001
NC	−1.55 ± 1.50	0.32 ± 0.07	0.50	1.51	<0.001
TOT	−1.89 ± 2.20	0.54 ± 0.10	0.57	2.21	<0.001

^1^ OM = omental fat; MES = mesenteric fat; PR = perirenal fat; ORG = organ fat (heart, liver, and lungs); CARC = carcass fat; NC = non-carcass fat; TOT, total fat depot (TOT = CARC + NC).

**Table 5:** Multiple regression equations and coefficient of determination (*R*^2^) and residual standard deviation (RSD) values for estimates of the different fat depot weights (kg) and the total weight of all the fat depots combined (y variables) based on the pre-slaughter measurement values body weight (BW); lumbar and sternal body condition scores (BCSl and BCSs, respectively); heart girth (HG); and lumbar, sternal, and perirenal fat thickness depths measured by ultrasound (FTUSl, FTUSs, and FTUSpr, respectively) (× variables).

Step	Dependent Variable (y) ^1^	Independent Variables (x)	Intercept ± Standard Error ^2^	b ± Standard Error	*R* ^2^	RSD
1	OM	FTUSpr	−7.77 ± 2.85	2.10 ± 0.27	0.79	0.65
2		HG		0.08 ± 0.03	0.85	0.57
1	MES	FTUSpr	−2.43 ± 1.13	0.35 ± 0.11	0.46	0.26
2		HG		0.03 ± 0.01	0.62	0.23
1	PR	FTUSpr	−1.66 ± 0.50	0.97 ± 0.11	0.84	0.26
2		BW		0.02 ± 0.01	0.88	0.23
1	ORG	FTUSl	−0.21 ± 0.15	0.07 ± 0.03	0.09	0.19
2		BW		0.004 ± 0.003	0.40	0.07
1	CARC	FTUSpr	−7.63 ± 1.14	0.80 ± 0.23	0.47	1.09
2		BW		0.09 ± 0.02	0.79	0.70
3		BCSl		2.25 ± 0.47	0.92	0.46
1	NC	FTUSpr	−18.21 ± 3.57	2.59 ± 0.36	0.70	1.25
2		HG		0.19 ± 0.04	0.89	0.76
3		BCSl		1.32 ± 0.75	0.91	0.71
1	TOT	FTUSpr	−15.80 ± 2.85	3.22 ± 0.58	0.62	2.28
2		BW		0.22 ± 0.05	0.86	1.44
3		BCSl		3.79 ± 1.18	0.92	1.14

All regressions are significant at *p* < 0.05. ^1^ OM = omental fat; MES = mesenteric fat; PR = perirenal fat; ORG = organ fat (heart, liver, and lungs); CARC = carcass fat; NC = non-carcass fat; TOT = total fat depot (TOT = CARC + NC). ^2^ The intercept is the same within each group of equations predicting the same dependent variable.

**Table 6:** Multiple regression equations and coefficient of determination (*R*^2^) and residual standard deviation (RSD) values for estimates of the different fat depot weights (kg) and the total weight of all the fat depots combined (y variables) based on the post-slaughter measurement values hot carcass weight (HCW), empty body weight (EBW), and lumbar and sternal fat thickness depths measured by a digital caliper (FTDCl and FTDCs, respectively) (x variables).

Step	Dependent Variable (y) ^1^	Independent Variable(s) (x)	Intercept ± Standard Error ^2^	b ± Standard Error	*R* ^2^	RSD
1	OM	EBW	−4.46 ± 1.31	0.09 ± 0.03	0.47	0.95
2		FTDCs		0.08 ± 0.05	0.52	0.92
1	MES	EBW	−0.86 ± 0.30	0.04 ± 0.01	0.61	0.21
1	PR	EBW	−2.16 ± 0.60	0.04 ± 0.02	0.46	0.44
2		FTDCs		0.04 ± 0.02	0.53	0.43
1	ORG	EBW	−0.54 ± 0.24	0.01 ± 0.005	0.23	0.17
2		FTDCl		0.11 ± 0.07	0.32	0.16
1	CARC	HCW	−3.83 ± 0.55	0.36 ± 0.07	0.82	0.57
2		FTDCs		0.12 ± 0.02	0.91	0.40
3		EBW		-0.06 ± 0.04	0.92	0.38
1	NC	EBW	−6.70 ± 1.58	0.19 ± 0.04	0.68	1.20
2		FTDCs		0.14 ± 0.06	0.74	1.12
1	TOT	HCW	−10.44 ± 2.00	0.60 ± 0.10	0.74	1.73
2		FTDCs		0.27 ± 0.08	0.83	1.41

All regressions are significant at *p* < 0.05. ^1^ OM = omental fat; MES = mesenteric fat; PR = perirenal fat; ORG = organ fat (heart, liver, and lungs); CARC = carcass fat; NC = non-carcass fat; TOT = total fat depot (TOT = CARC + NC). ^2^ The intercept is the same within each group of equations predicting the same dependent variable.

## Data Availability

The data presented in this study are available on request from the corresponding author.
